# Treatment with KCL‐286, a first‐in‐class retinoic acid receptor‐β (RARβ) agonist, ameliorates neuronal DNA damage and inflammation in a mouse model of Alzheimer's disease

**DOI:** 10.1002/2211-5463.70284

**Published:** 2026-07-08

**Authors:** Natasha Hill, Hassan Yousef Othman AlMuallim, Elouisa Maddock, Carl Hobbs, Earl Clarke, Maria B. Goncalves, Jonathan P. T. Corcoran

**Affiliations:** ^1^ Neuroscience Drug Discovery Unit, Wolfson Sensory, Pain and Regeneration Centre King's College London UK

**Keywords:** Alzheimer's disease, DNA double‐strand breaks, DNA repair, glial activation, neuroinflammation, RARβ agonist

## Abstract

Alzheimer's disease (AD) is a complex, multifactorial neurodegenerative disorder for which effective disease‐modifying therapies remain limited. Accumulation of neuronal DNA double‐strand breaks (DSBs) is an early pathological event that contributes to genomic instability and neuronal vulnerability in AD. Therapeutic strategies that enhance DNA repair may therefore be of considerable interest. Here, using the Tg2576 mouse model of AD, we show that treatment with KCL‐286, a selective retinoic acid receptor‐β (RARβ) agonist, reduces neuronal DNA damage. KCL‐286 enhances DSB repair in neurons, in part through upregulation of the DNA repair factor BRCA1, while also attenuating neuroinflammatory activation. In addition, KCL‐286 normalises microglial and astrocytic morphology, consistent with reduced pathological glial activation. Together, these findings demonstrate that selective RARβ activation ameliorates neuronal DNA damage and neuroinflammation in a mouse model of AD, supporting further investigation as a potential disease‐modifying therapeutic strategy.

## Graphical abstract

Repair of neuronal DNA damage in Alzheimer's disease by KCL‐286. (A) Amyloid‐β oligomers and plaques impair neuronal DNA repair pathways, leading to DNA double‐strand breaks and glial activation. (B) KCL‐286 activates RARβ/RXR signalling via retinoic acid response elements (RAREs), associated with increased BRCA1 expression, enhanced DNA repair and promotion of glial resolution.

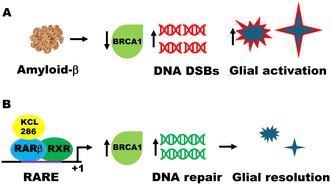

**Abbreviations**
A.U: arbitrary units
AD: Alzheimer's disease
AEs: adverse events
ANOVA: analysis of variance
APP: amyloid precursor protein
Aβ: amyloid‐beta
BRCA1: breast cancer type 1 susceptibility protein
CNS: central nervous system
DAPI: 4',6‐diamidino‐2‐phenylindole
DMSO: dimethyl sulfoxide
DSB: double‐strand break
dw: deionised water
FI: fluorescent intensity
GFAP: glial fibrillary acidic protein
GSK3β: Glycogen synthase kinase‐3 beta
HDAC1: Histone deacetylase 1
i.p.: intraperitoneal
Iba1: Ionised calcium‐binding adaptor molecule 1
IMS: industrialised methylated spirit
NeuN: neuronal nuclear protein
NFTs: neurofibrillary tangles
PARP1: Poly(ADP‐ribose) polymerase 1
PBS: phosphate‐buffered saline
POC: proof‐of‐concept
PS1/2: presenilin 1/2
pTau: phosphorylated Tau
RA: retinoic acid
RAR: retinoic acid receptor
RARE: retinoic acid receptor response element
ROI: region of interest
RXR: retinoid X receptor
SCI: spinal cord injury
SEM: standard error of the mean
TNF‐α: Tumour Necrosis Factor‐alpha
Veh: vehicle
WT: wild‐type
γH2AX: phosphorylated histone H2AX

Dementias are projected to increase to 153 million cases by 2050, with Alzheimer's disease (AD) accounting for most diagnoses [1]. In the United States alone, the number of AD cases is predicted to become 16.0 million cases by mid‐century [2]. Two pathological hallmarks of AD are extracellular plaques consisting of amyloid‐β (Aβ) and intracellular neurofibrillary tangles (NFTs), which are composed of phosphorylated Tau (pTau), but the underlying causes of the disease remain largely unknown [3]. Despite Aβ load being a poor indicator of disease progression [4], targeting Aβ with antibodies deaccelerates clinical decline, as evidenced by the two FDA‐approved therapies, Aducanumab and Lecanemab, which demonstrate measurable but limited clinical benefit [5]. Indeed, recent comprehensive analyses of clinical trial data have questioned whether Aβ‐directed therapies produce meaningful clinical improvement, particularly in patients with mild cognitive impairment or early dementia [6].

Given the multifactorial nature of AD, increasing attention has turned to other pathways known to be involved in AD, including neuroinflammation, mitochondrial dysfunction, neurotransmitter deficits and defects in autophagy, which has led to a number of clinical trials targeting these aspects [7]. To simultaneously target multiple pathways, avoiding a poly‐pharmacology approach, an ideal strategy would target a master regulatory transcription factor, or transcription‐factor family, capable of coordinating gene expression across multiple disease‐relevant pathways. One such candidate is the retinoic acid receptor (RAR)/retinoid X receptor (RXR) family, which mediates RA signalling and has been implicated in AD [8]. Deficits in RA signalling due to a vitamin A‐deficient diet in adult rats lead to the deposition of Aβ in the brain [9, 10], and conversely, in mouse models of AD, RA reduced Aβ plaque load by modulating amyloid precursor protein (APP) processing, as well as preventing tau phosphorylation and inflammation [11, 12].

The principal challenge in therapeutically targeting RA signalling lies in the development of retinoid drugs with an appropriate selectivity profile that minimises off‐target effects. To address this, we have recently developed KCL‐286 (also known as C286), a RARβ agonist that enables engagement of the retinoic acid transcriptional programme through a defined receptor subtype. KCL‐286 has been shown to be safe in healthy human participants at doses encompassing the envisaged therapeutic window, extrapolated from proof‐of‐concept (POC) studies in rodent models of spinal cord injury (SCI) [13, 14, 15].

Targeting this master regulatory transcriptional axis is of particular interest given the substantial convergence of pathological pathways across neurodegenerative conditions, including neuroinflammation, mitochondrial dysfunction, impaired phagocytosis and neurotransmitter deficits [14, 15, 16]. One key downstream mechanism through which KCL‐286 counteracts neurodegeneration is the promotion of DNA double‐strand break (DSB) repair, mediated by the upregulation of breast cancer type 1 susceptibility protein (BRCA1) [17]. Notably, DSBs are significantly elevated in the brains of AD patients compared with non‐AD controls, and deficits in BRCA1 signalling have also been reported in AD [18, 19, 20].

Here, we demonstrate that in Tg2576 mice, which overexpress human APP harbouring the Swedish double mutation (K670N, M671L) under the control of the hamster prion protein promoter—resulting in increased production of human amyloid‐β (Aβ) and progressive plaque accumulation [21]—treatment with KCL‐286 ameliorates neuronal DNA DSB pathology and significantly attenuates neuroinflammatory activation. These effects are associated, at least in part, with the upregulation of the DNA repair factor BRCA1 and concomitant normalisation of glial activation states.

Together, this multifactorial mode of action in a neurodegenerative context supports further evaluation of KCL‐286 as a potential disease‐modifying therapeutic strategy for AD, either as a monotherapy or in rational combination with existing approaches, such as Aβ‐directed immunotherapies, applied at defined stages of disease progression.

## Methods and materials

### Ethics statement, study approval

All animal care and experimental procedures complied with the Animals (Scientific Procedures) Act 1986 of the UK Parliament, Directive 2010/63/EU of the European Parliament, and the Guide for the Care and Use of Laboratory Animals published by the US National Institutes of Health (NIH Publication No. 85–23, revised 1996). All animal experiments were approved by King's College London (project licence: 7009067) and conducted in accordance with institutional guidelines and the ARRIVE 2.0 guidelines.

### Animal treatments and brain fixing

Male Tg2576 mice on a 129S6 background and respective wild‐type (WT) littermate mice were purchased from Taconic Farms (Germantown, NY, USA). Mice were maintained on a 12/12‐h light–dark cycle at 20–22 °C and given food and water *ad libitum*. Mice (*n* = 3 per group) were treated by intraperitoneal (i.p.) injections of either vehicle (Veh; 80% dimethyl sulfoxide, DMSO, in distilled water) or 1 mg/kg with KCL‐286 three times a week from 15 to 18 months of age. They were anaesthetised by i.p. injection of pentobarbitone, and transcardially perfused with heparinised saline. Brains were removed and half hemispheres were postfixed with 4% paraformaldehyde (in 0.1 m phosphate buffer) for at least two days at room temperature. Brain tissue was embedded in paraffin wax. The mice brains were coded and remained blinded to which groups they belonged to until completion of data analysis.

### Immunohistochemistry and antibodies

For a given antibody, all sections were processed at the same time. Immunohistochemistry was carried out as previously described [15]. Six μm brain sections were cut with a rotary microtome and placed in a water bath containing warm water. They were then mounted onto SuperFrost Plus slides. sections were first dewaxed in xylene and 100% Industrial Methylated Spirit (IMS), then heated in citric acid (10 mM, pH = 6), until boiling, then washed under a running tap for 5 min. Pwax sections were washed three times for 5 min each in phosphate‑buffered saline (PBS) before incubation with the primary antibody in PBS‐0.02% Tween at 4 °C overnight. The primary antibody was removed by washing three times for 5 min each in PBS. They were incubated in the secondary antibody for 1 h at room temperature (RT) in PBS‐0.02% Tween and then washed in PBS three times for 5 min. Antibodies used were: rabbit polyclonal anti‐BRCA1 (Abcam plc, Cambridge, UK; 1:250); rabbit monoclonal anti‐GFAP (glial fibrillary acidic protein, Sigma Aldrich, St. Louis, MO, USA; 1:200), rabbit polyclonal anti‐Iba1 (ionised calcium‑binding adaptor molecule 1, DAKO, Agilent Technologies, Glostrup, Denmark; 1:1000); mouse monoclonal anti‐NeuN (Sigma, 1:500), rabbit monoclonal anti‐γH2AX (phosphorylated histone H2AX, Abcam, 1:500). Secondary antibodies used were Donkey anti‐mouse (Alexa FluorTM 488; 1:500; ThermoFisher, Waltham, MA, USA) or Goat anti‐mouse (Alexa FluorTM 488; 1:500; ThermoFisher, USA), Donkey anti‐rabbit (Alexa FluorTM 594; 1:500; ThermoFisher, USA), and 4′,6‑diamidino‑2‑phenylindole (DAPI) (Sigma‐Aldrich, USA) used to stain cell nuclei.

### Confocal microscopy and postprocessing of images

The sections were visualised using a Zeiss LSM 710 multichannel confocal microscope employing laser scanning technology (2020). A 40x oil‐immersion objective from Carl Zeiss was employed. The gain, laser power and pinhole size were optimised through the ZEN 2.5 (blue edition) software from Carl Zeiss microscopy GmbH (2018) and remained uniform. The digital offset and gain were not altered. Three separate images of each region of interest (ROI) were taken for each mouse brain section. Adobe Photoshop CS4 (version 11) was employed to standardise the brightness and contrast of each image to ensure uniformity of the background signal.

### Quantification

All analyses were performed using ImageJ (version 1.54g, Java 1.8.0_345, 64‑bit).

#### γH2AX

Colour channels were split and the red channel corresponding to anti‐γH2AX staining was selected. A consistent manual threshold was applied across all samples. Background signal was assessed by measuring mean grey value. Ten neuronal nuclear protein (NeuN) positive neurons co‐labelled for γH2AX were selected per section, and cell bodies were manually delineated using the freehand selection tool. Measurements (Analyse > Measure) included mean grey value, area, and integrated density. The average mean grey value was calculated for each region for each animal.

#### BRCA1

Colour channels were split and the red channel corresponding to BRCA1 staining was selected. A consistent manual threshold was applied across all samples. BRCA1‑positive puncta were quantified using the Analyse Particles function (Analyse > Analye Particles) with a uniform size threshold and circularity set to 0.00–1.00. The total number of particles above this size threshold were counted as the number of BRCA1‐positive punctae in each image; the average number of BRCA1‐positive punctae was calculated for each region for each animal.

### GFAP and Iba1 area‐fraction analysis (glial burden)

Colour channels were split, and the green channel was analysed for GFAP staining, while the red channel was analysed for Iba1 staining. Images were converted to 8‐bit greyscale, and a consistent manual threshold was applied across all images to identify positive signal. Measurements included mean grey value, minimum and maximum grey values, and area fraction. As the ROI was uniform across all images, GFAP‐ and Iba1‐positive signal was quantified as area fraction (% of ROI area) rather than absolute area. Area‐fraction values were averaged per region for each animal.

### Microglial morphology: Iba1 soma area

Microglial activation state was assessed by quantifying Iba1‐positive soma area. Individual microglial somata were identified based on Iba1 staining and nuclear localisation, and cell bodies were manually outlined using ImageJ. Soma area was calculated for each cell.

For each animal, soma area was quantified from approximately 5–11 Iba1‐positive microglia per section. Soma area was averaged per region for each animal.

### Astrocyte morphology: GFAP Process thickness

Astrocyte morphology was assessed by measuring GFAP‐positive process thickness. Within ROI, astrocytic processes were identified and measured perpendicular to the process axis using ImageJ. Approximately 5–10 astrocytes were sampled per section, with one or more clearly identifiable processes measured per astrocyte where possible. Measurements were averaged across regions for each animal. This morphology‐based metric provides a sensitive measure of hypertrophic astrogliosis that is independent of overall GFAP expression level.

### Statistical analysis

All statistical analyses were performed using SPSS. Data were analysed at the animal level, and values are presented as mean ± standard error of the mean (SEM). Normality and homogeneity of variance were assessed prior to statistical testing. For datasets violating assumptions of homogeneity of variance (Levene's test), including γH2AX fluorescence intensity (FI), Iba1 area fraction and GFAP area fraction, Kruskal–Wallis tests were used, followed by Mann–Whitney U post hoc tests. Significance levels for post hoc Mann–Whitney comparisons were adjusted using the Bonferroni correction for multiple testing. BRCA1‐positive nuclear punctae were analysed using one‐way analysis of variance (ANOVA), followed by Tukey's post hoc tests to compare differences across mouse groups. Morphological measurements, including Iba1 soma area and GFAP process thickness, were analysed using one‐way ANOVA followed by Holm–Sidak post hoc tests. Statistical significance was defined as: *P* ≤ 0.05 (*), *P* ≤ 0.01 (**), *P* ≤ 0.001 (***). Bar graphs were generated using SPSS.

## Results

### KCL‐286 reduces DNA double‐stranded breaks in the Tg2576 mouse

We first assessed whether KCL‐286 could ameliorate neuronal DNA DSBs in male Tg2576 mice, which were treated with 1 mg/kg KCL‐286 or Veh by i.p. injection three times per week from 15 to 18 months of age. Histone H2AX is phosphorylated at sites of DNA DSBs, forming γH2AX, and detection of γH2AX foci is widely used as a marker of DNA damage [22].

**Fig. 1:**
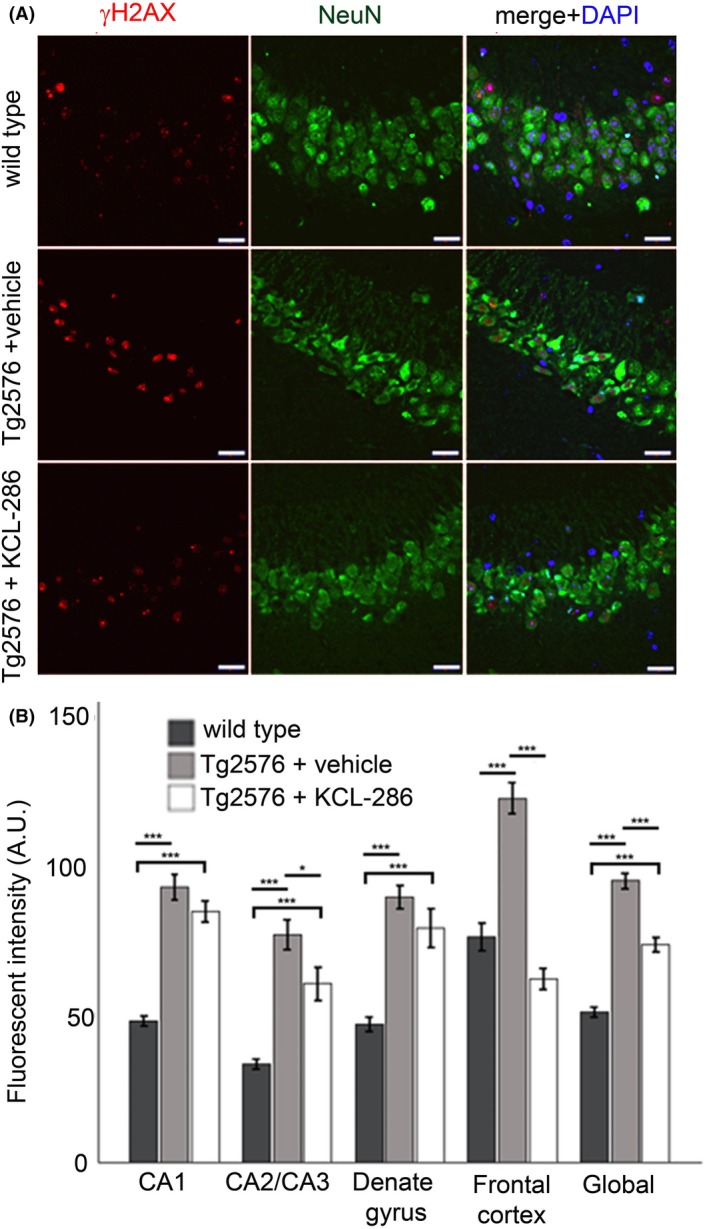
KCL‐286 reduces nuclear γH2AX expression in Tg2576 mouse neurons. (A) Immunostaining for γH2AX (red), NeuN (green) and DAPI (blue) in the CA2/CA3 region of the hippocampus. γH2AX co‐localises with NeuN‐positive neuronal nuclei. (B) Quantification of γH2AX FI, expressed in arbitrary units (A.U.), within NeuN‐positive neurons across hippocampal and cortical regions and in global analysis. γH2AX FI is significantly higher in Veh‐treated Tg2576 mice compared with WT mice across all regions and globally, and significantly higher than in KCL‐286‐treated Tg2576 mice in the CA2/CA3 region of the hippocampus, the frontal cortex and globally. Data are presented as mean ± SEM. Statistical analysis was performed using Kruskal–Wallis tests with Mann–Whitney U post hoc tests and Bonferroni correction for multiple comparisons; *P ≤ 0.05, **P ≤ 0.01, ***P ≤ 0.001. *n* = 3 mice per group, with three images analysed per region per animal. Scale bar, 20 μm.

Veh‐treated Tg2576 mice exhibited significantly higher γH2AX FI in NeuN‐positive neurons compared with WT littermates across all regions examined, including hippocampal subfields and frontal cortex (Fig. 1A,B). When data from all regions were collated, γH2AX FI differed significantly between experimental groups (Kruskal–Wallis, H(2, *n* = 639) = 161.92, *P* < 0.001; Fig. 1B). Tg2576 mice treated with KCL‐286 showed a significant reduction in neuronal γH2AX FI globally (mean = 73.74 ± 35.32 A.U.) compared with veh‐treated Tg2576 mice (mean = 95.11 ± 38.88 A.U.; Mann–Whitney U test with Bonferroni correction, *P* < 0.001), indicating attenuation of neuronal DNA damage by KCL‐286 (Fig. 1B). Despite this reduction, γH2AX FI in KCL‐286‐treated Tg2576 mice remained significantly elevated relative to WT controls (mean = 50.71 ± 23.94 A.U.; *P* < 0.001).

Notably, the magnitude of γH2AX reduction following KCL‐286 treatment was region‐dependent. While significant decreases were observed in the frontal cortex and in global analyses, hippocampal subfields exhibited more modest reductions that did not uniformly reach significance (Fig. 1B). This pattern is consistent with the greater vulnerability and more advanced pathology of hippocampal neurons in the Tg2576 model [23], which may limit the capacity for pharmacological rescue of DNA damage at this disease stage compared with cortical regions.

### KCL‐286 induces DNA repair protein BRCA1 neuronal expression in the Tg2576 mouse

**Fig. 2:**
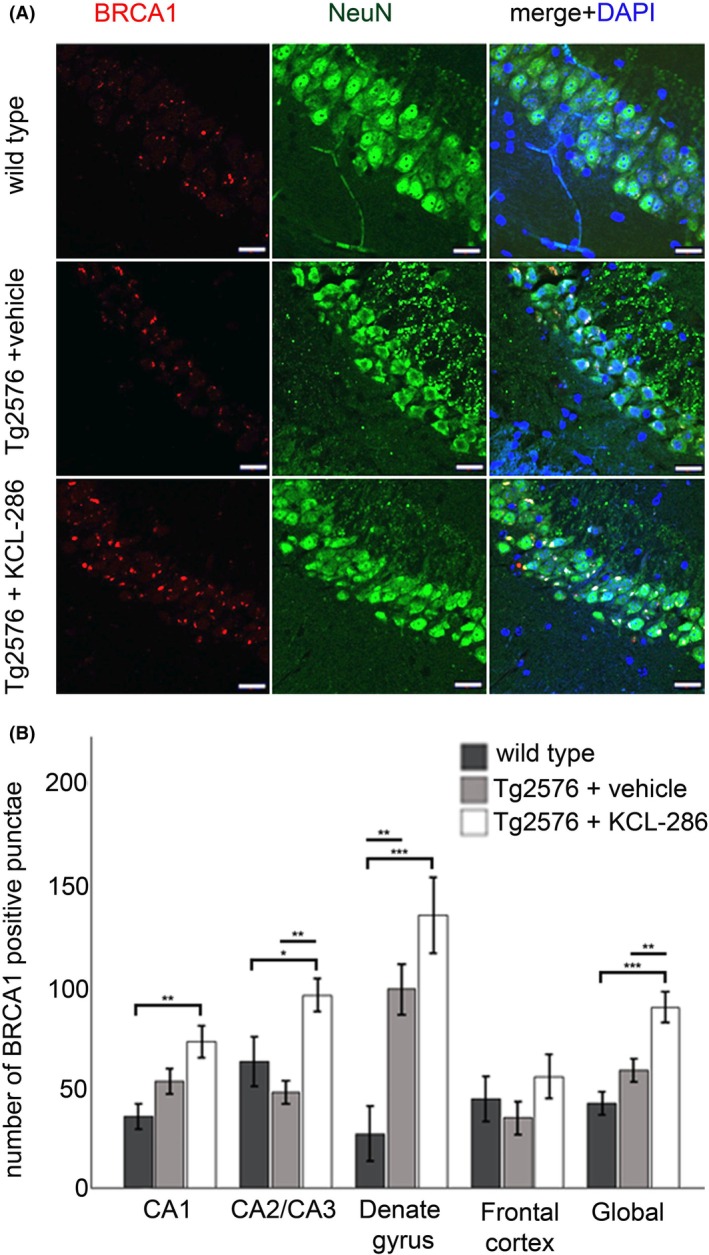
KCL‐286 induces expression of the DNA damage repair factor BRCA1 in Tg2576 mouse neurons. (A) Immunostaining for BRCA1 (red), NeuN (green) and DAPI (blue) in the CA2/CA3 region of the hippocampus. BRCA1 colocalises with NeuN‐positive neuronal nuclei, and the number of BRCA1‐positive punctae is increased in KCL‐286‐treated mice compared with WT and Veh‐treated Tg2576 mice. (B) Quantification of the number of BRCA1‐positive punctae per NeuN‐positive neuron in each region of interest and in global analysis. Neurons from KCL‐286‐treated Tg2576 mice exhibit significantly greater numbers of BRCA1‐positive punctae compared with Veh‐treated mice in the CA2/CA3 region and globally. Data are presented as mean number of BRCA1‐positive punctae ± SEM. Statistical analysis was performed using one‐way ANOVA with Tukey post hoc tests; *P ≤ 0.05, **P ≤ 0.01, ***P ≤ 0.001. *n* = 3 mice per group, with three images analysed per region per animal. Scale bar, 20 μm.

We next asked whether the drug could upregulate proteins known to be involved in DNA repair. One candidate is BRCA1 that has been linked to AD pathology, with both Aβ and ptau contributing to its decreased expression and function [18, 24, 25]. We have already shown that KCL‐286 significantly increases the expression of BRCA1 in a neuropathic pain model [17]; therefore, we assessed whether KCL‐286 could similarly upregulate the levels of BRCA1 expression in the Tg2576 mouse. We found that BRCA1 co‐localised with NeuN and was particularly located at the perinuclear region (Fig. 2A,B). An ANOVA test revealed that the number of BRCA1‐positive punctae was significantly different across the groups globally (*F* (2,104) = 13.845, *P* < 0.001) and Tukey post hoc tests showed that neurons in KCL‐treated mice (mean = 89 ± 46) had significantly more punctae than both WT (mean = 42 ± 34, *P* < 0.001) and Veh‐treated (mean = 58 ± 35, *P* = 0.003) (Fig. 2B). KCL‐286‐treated had significantly more punctae in neurons in the CA2/CA3 region of the hippocampus than Veh‐treated mice (*P* = 0.002) (Fig. 2B).

### KCL‐286 differentially modulates glial marker expression in the Tg2576 mouse

Neuroinflammation is a physiological process essential for the clearance of pathogens, cellular debris, and protein aggregates. However, in AD, neuroinflammatory responses become persistent and contribute to neuronal dysfunction and degeneration [26]. Given that KCL‐286 has been shown to modulate inflammatory pathways in models of neuropathic pain and spinal cord contusion [15, 17], we investigated whether KCL‐286 exerts a similar effect in the Tg2576 mouse model of AD.

One hallmark of neuroinflammation in AD is the chronic activation of microglia, often accompanied by cellular hypertrophy and increased expression of inflammatory markers [27]. Iba1 is a microglia‐specific protein that is upregulated upon activation [28]; therefore, anti‐Iba1 immunofluorescence was used to quantify microglial activation across hippocampal and cortical regions.

**Fig. 3:**
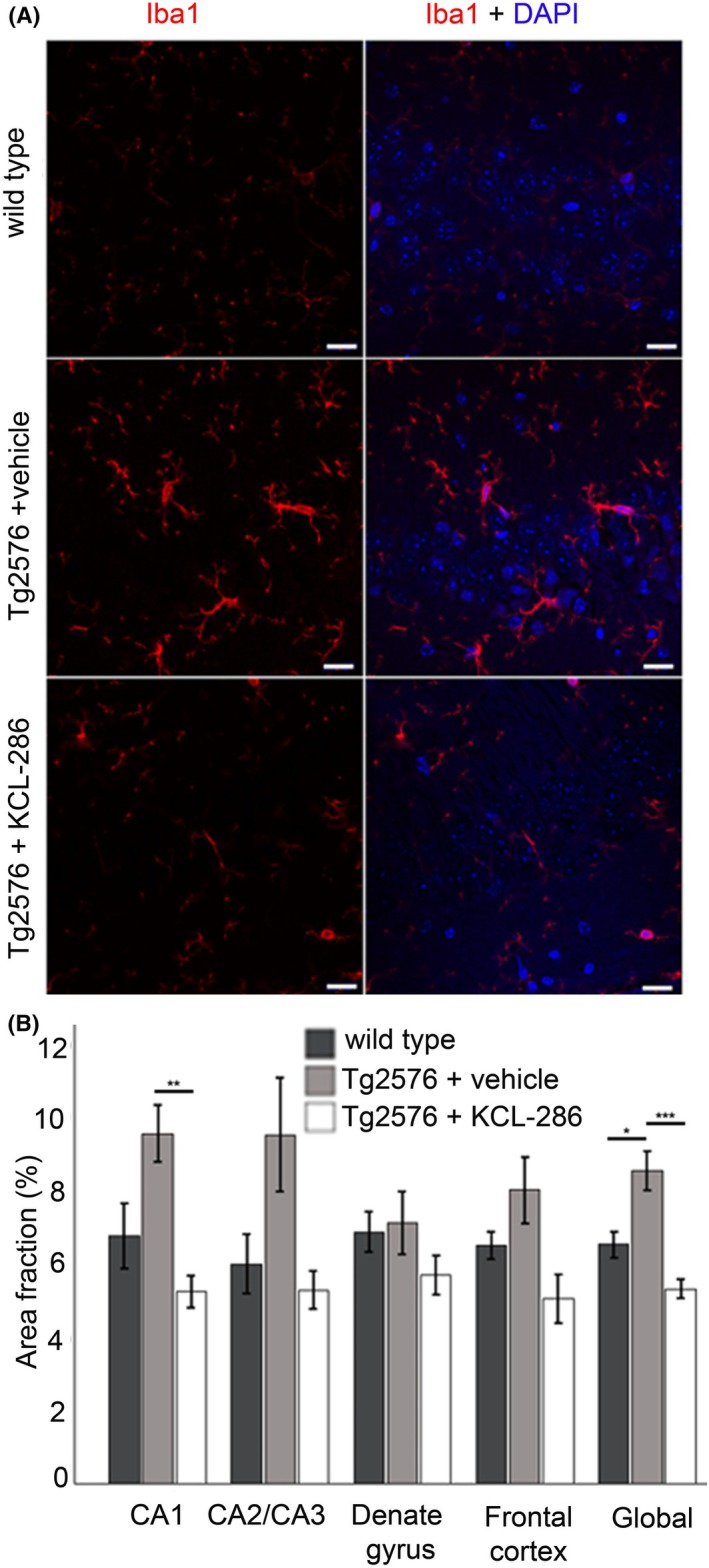
KCL‐286 reduces Iba1 expression in Tg2576 mice. (A) Iba1 expression in the CA2/CA3 region of the hippocampus. Immunostaining for Iba1 (red) and DAPI (blue). Iba1‐positive area is visibly increased in Veh‐treated Tg2576 mice compared with WT and KCL‐286‐treated mice. (B) Quantification of Iba1‐positive area fraction (%) in each region of interest and in global analysis. Iba1 area fraction is significantly lower in KCL‐286‐treated mice compared with Veh‐treated Tg2576 mice in the CA1 region of the hippocampus and globally. Data are presented as mean area fraction ± SEM. Statistical analysis was performed using Kruskal–Wallis tests followed by Mann–Whitney U post hoc tests with Bonferroni correction for multiple comparisons; *P ≤ 0.05, **P ≤ 0.01, ***P ≤ 0.001. n = 3 mice per group, with three images analysed per region per animal. Scale bar, 20 μm.

Globally, Iba1 area fraction differed significantly between experimental groups (H(2, *n* = 105) = 21.761, *P* < 0.001; Fig. 3A,B). Post hoc Mann–Whitney tests with Bonferroni correction revealed that Veh‐treated Tg2576 mice exhibited significantly greater Iba1 area fraction compared with WT animals (8.56 ± 0.53% vs. 6.54 ± 0.36%, *P* = 0.05) and compared with KCL‐286‐treated Tg2576 mice (5.32 ± 0.26%, *P* < 0.001). Despite consistently lower Iba1 area fraction in KCL‐286‐treated mice across all regions examined, statistically significant regional reductions were confined to the CA1 region of the hippocampus (*P* = 0.004; Fig. 3B).

**Fig. 4:**
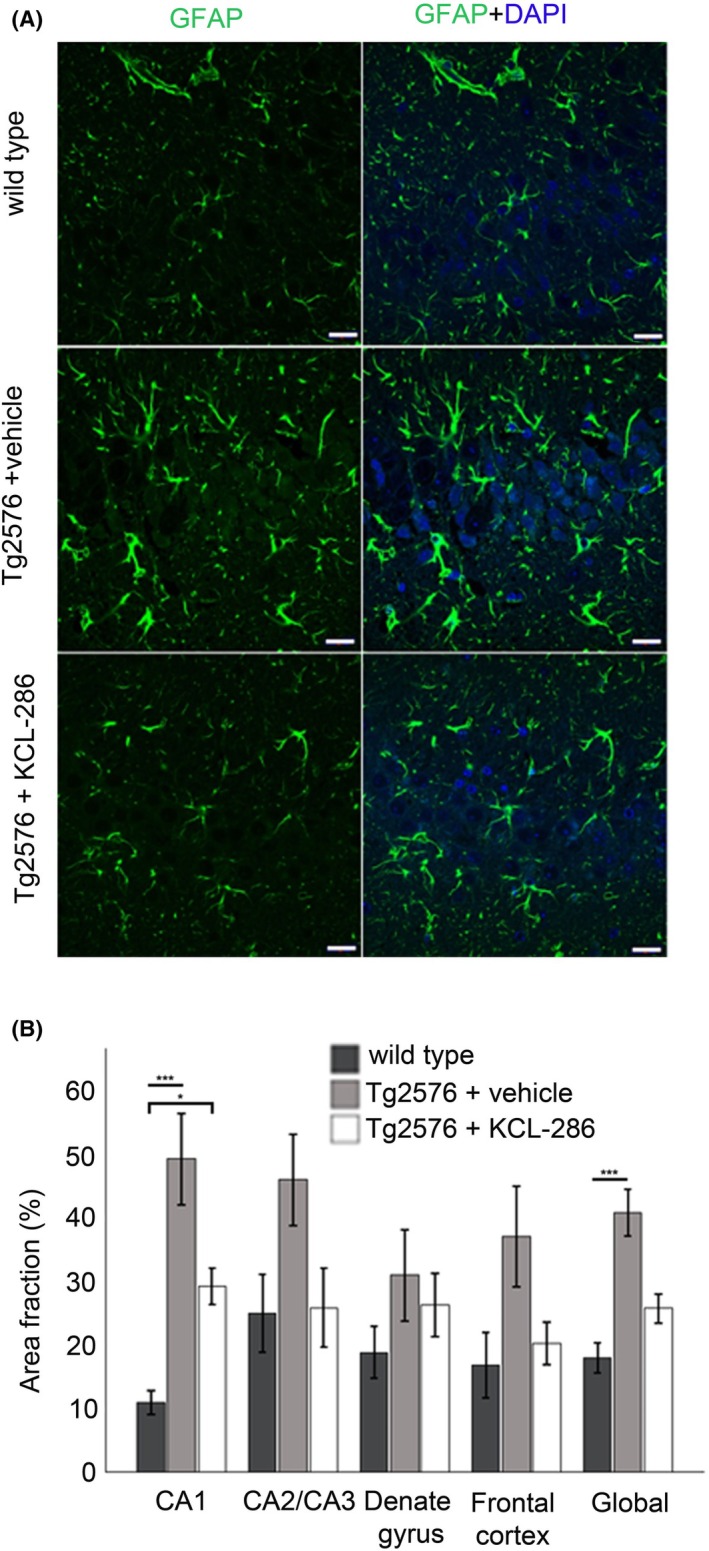
KCL‐286 does not significantly reduce astrogliosis. (A) GFAP expression in the CA2/CA3 region of the hippocampus. Immunostaining for GFAP (green) and DAPI (blue). (B) Area fraction (%) of GFAP staining in each region of interest and globally for each group. While the mean area fraction (%) of GFAP staining in KCL‐286‐treated mice is substantially lower than in Veh‐treated mice in all regions except the dentate gyrus, no statistical significance was reached. Data are presented as mean area fraction ± SEM. Kruskal‐Wallis with Mann–Whitney post hoc tests with Bonferroni correction for multiple tests, *P ≤ 0.05, ***P ≤ 0.001. *n* = 3 mice per group, with three images analysed per region per animal. Scale bar, 20 μm.

Reactive astrogliosis represents a second hallmark of neuroinflammation in AD, characterised by astrocytic hypertrophy and increased expression of intermediate filament proteins, particularly in the vicinity of Aβ plaques [29]. Astrocytic reactivity was therefore evaluated using GFAP immunostaining, a widely used marker of reactive astrocytes. GFAP area fraction differed across groups at the global level (H(2, *n* = 106) = 21.693, *P* < 0.001; Fig. 4A,B), with significant differences observed between WT and Veh‐treated Tg2576 mice (*P* < 0.001). While KCL‐286‐treated mice exhibited lower GFAP area fraction than Veh‐treated animals across multiple regions, these reductions did not reach statistical significance following correction for multiple comparisons (Fig. 4B).

### KCL‐286 attenuates glial activation state through morphological normalisation in the Tg2576 mouse

**Fig. 5:**
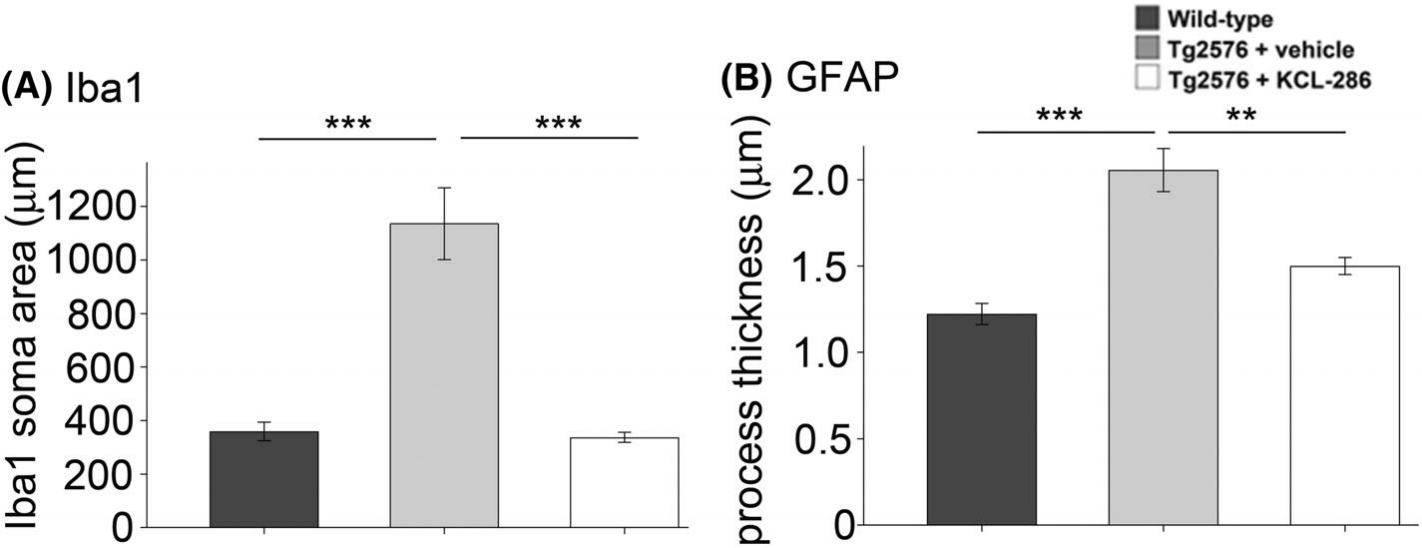
KCL‐286 normalises glial morphology. (A) Quantification of Iba1‐positive microglial soma area reveals marked cellular hypertrophy in Veh‐treated Tg2576 mice compared with WT animals. KCL‐286 treatment significantly reduces microglial soma area, restoring values to those observed in WT mice. (B) Quantification of GFAP‐positive astrocyte process thickness. Veh‐treated Tg2576 mice exhibit marked astrocytic hypertrophy compared with WT animals. KCL‐286 treatment significantly reduces GFAP process thickness towards WT levels. Data are presented as mean ± SEM. Morphological measures were analysed using one‐way ANOVA with Holm–Sidak post hoc tests; **P ≤ 0.01, ***P ≤ 0.001. *n* = 3 mice per group, with three images analysed per region per animal.

Given that area‐fraction measures alone do not distinguish between changes in glial abundance and cellular activation state [29, 30], microglial morphology was additionally assessed using cell‐level measurements. Veh‐treated Tg2576 mice exhibited pronounced microglial hypertrophy, reflected by a threefold increase in Iba1‐positive soma area compared with WT mice (one‐way ANOVA, F(2,6) = 31.735, *P* < 0.001; Fig. 5A). In contrast, KCL‐286 treatment significantly reduced microglial soma area relative to Veh‐treated Tg2576 mice (*P* = 0.001), restoring soma size to values indistinguishable from WT (*P* = 0.854). These findings indicate that KCL‐286 normalises microglial activation state through restoration of cell morphology, rather than simply reducing overall Iba1 expression or microglial presence.

Importantly, direct quantification of astrocyte morphology revealed a significant effect of treatment on GFAP‐positive process thickness (one‐way ANOVA, F(2,6) = 24.879, *P* = 0.001; Fig. 5B). Veh‐treated Tg2576 mice displayed marked astrocytic hypertrophy compared with WT controls (*P* = 0.001), while KCL‐286 treatment significantly reduced process thickness relative to Veh‐treated mice (*P* = 0.007). Astrocyte process thickness in KCL‐286‐treated mice did not differ significantly from WT levels (*P* = 0.061), consistent with attenuation of reactive hypertrophy without over suppression.

Together, these results demonstrate that KCL‐286 reduces glial reactivity in Tg2576 mice primarily by normalising astrocytic and microglial morphology, rather than by fully suppressing glial marker expression or eliminating glial cells. Accordingly, KCL‐286 treatment was associated with attenuation of pathological glial activation states without evidence of glial depletion or loss of baseline marker expression.

## Discussion

Ageing is the single most powerful risk factor for the onset of dementias [31] and RA signalling is dampened with age [32], which could contribute to the loss of cellular resilience and resistance to stressors that permit the onset of pathology and progression to clinical symptomatology. One such stressor is the accumulation of DNA DSBs, which occur early in the pathology of AD [33, 34]. Therefore, it is of importance to identify drugs that can repair DNA. Here, we have shown the potential of the retinoid KCL‐286 as a therapeutic for AD; it is an RARβ agonist drug, has a blood brain barrier penetration of 1:1, a good PK profile [35] with target engagement demonstrated and no drug related AEs being reported in a phase I trial in human healthy participants [14, 15].

Using the Tg2576 mouse model, we found that KCL‐286 enhanced neuronal DNA double‐strand break (DSB) repair, in part through upregulation of the DNA repair factor BRCA1. While our analysis focussed on a single aspect of DNA repair, resolution of neuronal DSBs was also observed in the absence of increased BRCA1 expression, indicating that additional repair pathways are likely engaged. These may include ataxia telangiectasia mutated, DNA‐dependent protein kinase catalytic subunit, poly(ADP‐ribose) polymerase‐1, histone deacetylase‐1 and glycogen synthase kinase‐3β, all of which have been reported to be compromised in AD [36].

In other transgenic mouse models overexpressing human APP, BRCA1 expression is reduced relative to WT controls [24], highlighting important model‐ and disease‐stage‐specific differences in DNA repair capacity. These findings likely reflect a failure of DNA repair pathways in more advanced disease states. In contrast, in the Tg2576 mouse, we observe an apparent compensatory upregulation of BRCA1 in hippocampal neurons, likely reflecting an endogenous attempt to counteract accumulating DNA damage. This response is region‐specific and insufficient to fully prevent persistent DNA double‐strand breaks, suggesting that DNA repair mechanisms are stressed but not yet completely collapsed at this stage of disease.

Consistent with this interpretation, the more pronounced reduction in neuronal γH2AX observed in cortical regions compared with hippocampal subfields following KCL‐286 treatment likely reflects regional differences in disease severity, with hippocampal neurons exhibiting more advanced pathology [37] and a reduced capacity for DNA damage resolution at the time of intervention. This suggests that KCL‐286 is most effective when endogenous DNA repair pathways remain inducible.

An additional layer of complexity in AD is the interaction between tau pathology and DNA repair. PTau has been shown to sequester BRCA1 in the cytoplasm, preventing its translocation to the nucleus and thereby impairing DNA DSB repair [18, 20]. This mechanism is not present in the Tg2576 mouse, which lacks overt tau pathology. However, retinoids have previously been shown to inhibit tau phosphorylation through nongenomic mechanisms [12], raising the possibility that KCL‐286 may indirectly preserve BRCA1 nuclear function in tau‐dependent disease contexts. Whether similar effects occur in tau‐dependent AD models warrants further investigation.

A further consequence of DSB in AD is that they cause microglia activation [38], which can remove Aβ, but their overactivation can exacerbate neuronal damage and disease progression [39, 40, 41]. We do not know the factors involved in the reduction of inflammation; however, in AD, tumour necrosis factor‐α (TNFα) is markedly upregulated [42] and we had previously shown that KCL‐286 downregulates TNF‐α in acute nerve injuries [17] raising the possibility that similar signalling pathways may contribute to the modulation of glial activation observed in the Tg2576 mouse.

KCL‐286 treatment was associated with a normalisation of microglial morphology towards a WT‐like phenotype, without evidence of microglial depletion or nonspecific suppression of Iba1 expression. This pattern indicates that KCL‐286 modulates microglial activation state rather than reducing microglial abundance. Such modulation towards a more homeostatic phenotype is increasingly viewed as therapeutically favourable in neurodegenerative disease, where preserving essential microglial functions while limiting maladaptive activation may mitigate neuronal stress and disease progression [30].

Astrocytes are increasingly recognised as active contributors to neuroinflammation in AD rather than passive responders to pathology. Reactive astrogliosis is characterised by astrocytic hypertrophy, cytoskeletal remodelling, and altered homeostatic functions, including impaired glutamate uptake, dysregulated cytokine release and disruption of neuronal–glial metabolic coupling [23]. In the Tg2576 mouse, Veh treatment was associated with marked thickening of GFAP‐positive astrocytic processes, consistent with a hypertrophic reactive phenotype commonly observed in proximity to Aβ pathology [29]. KCL‐286 treatment significantly attenuated astrocytic process thickening without fully suppressing GFAP expression, indicating a reversal of pathological hypertrophy rather than loss of astrocytes or general inhibition of astrocytic function. This distinction is critical, as astrocytes play essential roles in synaptic support, neurotransmitter recycling, and maintenance of extracellular homeostasis, and excessive suppression of astrocytic activity may be detrimental [43].

Taken together, the parallel normalisation of microglial and astrocytic morphology suggests that KCL‐286 acts to dampen the overall neuroinflammatory glial milieu. These findings support the concept that selective attenuation of glial activation states while preserving baseline glial homeostatic functions may represent a therapeutically advantageous strategy in AD.

To date, six retinoidshave entered the clinic [44] of which two have been used in POC trials for AD but failed to yield clinical benefit and led to adverse events (AEs). Acitretin treatment was associated with increased inflammatory markers [45, 46], while bexarotene treatment led to elevated triglycerides [47]. Notably, both compounds are nonselective retinoid receptor agonists engaging multiple RAR and RXR subtypes simultaneously.

In contrast, selective activation of RARβ enables a more controlled and coherent transcriptional response. We have previously shown that RA signalling initiated by RARβ is required for effective nerve repair and adult neurogenesis [48, 49, 50]. These findings highlight receptor selectivity as a critical determinant of therapeutic outcome.

Deficits in RARβ signalling have been reported in multiple neurodegenerative disorders, including Parkinson's disease, motor neuron disease and Huntington's disease [51, 52, 53]. Given the shared features of the neurodegenerative milieu across trauma‐induced and progressive chronic neurodegenerative conditions, including sustained neuroinflammation, glial activation and impaired repair processes, we propose that a defined sequence of retinoid receptor activation, initiated by RARβ signalling, may promote adaptive repair responses in the CNS [15, 16].

Further support for a conserved role of RARβ signalling in neural repair comes from recent evidence that osteopontin promotes Aβ clearance [54], a pathway also induced by KCL‐286 [15]. Osteopontin induction has additionally been observed in acute nerve injury models in which Aβ accumulation occurs [55], suggesting that overlapping molecular programmes may operate across acute injury and chronic neurodegeneration.

AD is a multifactorial disorder of unknown aetiology, and master regulators capable of coordinating multiple pathogenic pathways have been proposed as promising disease‐modifying targets [56]. RARβ, which functions as a transcriptional regulator of central nervous system axogenesis and repair [16], may represent such a regulator. The transcriptional and cellular pathways activated by KCL‐286 in regeneration models overlap substantially with mechanisms implicated in AD pathology, including neuroinflammation, chemokine signalling, extracellular matrix remodelling, phagocytosis and disrupted synaptic signalling [15]. These parallels suggest that injury‐associated repair programmes may recapitulate key processes relevant to AD progression.

Pathological accumulation of Aβ has also been shown to impair endogenous RA synthesis in the adult brain [12], potentially exacerbating disease‐associated dysfunction and limiting the efficacy of Aβ‐targeted therapies used in isolation [57]. Combination approaches pairing Aβ‐lowering strategies with selective RARβ agonism may therefore amplify therapeutic benefit by restoring broader homeostatic signalling networks. Consistent with prior nerve injury models, KCL‐286 may initiate feedback loops involving RA production that support endogenous repair programmes [50].

Collectively, these findings demonstrate that KCL‐286 ameliorates neuronal DNA damage and attenuates neuroinflammatory activation in the Tg2576 mouse model of AD. Together with its favourable human safety profile, the data support further investigation of selective RARβ agonism as a therapeutic strategy for modifying pathogenic processes associated with AD.

## Conflict of interest

MG and JC have a matter of composition patent for KCL‐286.

## Author contributions

JC conceived and designed the study. NH, HA, EM and CH acquired the data. MG and EC conducted the rodent studies. NH, HA, EM, JC and MG analysed and interpreted the data. NH, HA, EM, JC and MG wrote the manuscript.

## Data Availability

The data that support the findings of this study are available from the corresponding author upon reasonable request.
